# Small variations in reaction conditions tune carbon dot fluorescence†

**DOI:** 10.1039/d2nr01306a

**Published:** 2022-03-25

**Authors:** Teodoro Garcia-Millan, Thomas A. Swift, David J. Morgan, Robert L. Harniman, Benjamin Masheder, Stephen Hughes, Sean A. Davis, Thomas A. A. Oliver, M. Carmen Galan

**Affiliations:** School of Chemistry, University of Bristol Cantock's Close Bristol BS8 1TS UK tom.oliver@bristol.ac.uk m.c.galan@bristol.ac.uk; Cardiff Catalysis Institute, Cardiff University Park Place Cardiff CF10 3AT UK; HarwellXPS, – ESPRC National Facility for XPS, Research Complex at Harwell (RcAH) Didcot Oxon OX11 0FA UK; DST Innovations Ltd, Unit 6a Bridgend Business Centre Bennett Street Bridgend CF31 3SH UK

## Abstract

The development of robust and reproducible synthetic strategies for the production of carbon dots (CDs) with improved fluorescence quantum yields and distinct emission profiles is of great relevance given the vast range of applications of CDs. The fundamental understanding at a molecular level of their formation mechanism, chemical structure and how these parameters are correlated to their photoluminescence (PL) properties is thus essential. In this study, we describe the synthesis and structural characterization of a range of CDs with distinct physico-chemical properties. The materials were prepared under three minutes of microwave irradiation using the same common starting materials (d-glucosamine hydrochloride 1 and ethylenediamine 2) but modifying the stoichiometry of the reagents. We show that small variation in reaction conditions leads to changes in the fluorescent behaviour of the CDs, especially in the selective enhancement of overlapped fluorescence bands. Structural analysis of the different CD samples suggested different reaction pathways during the CD formation and surface passivation, with the latter step being key to the observed differences. Moreover, we demonstrate that these materials have distinct reversible response to pH changes, which we can be attribute to different behaviour towards protonation/deprotonation events of distinct emission domains present within each nanomaterial. Our results highlight the importance of understanding the reaction pathways that lead to the formation of this carbon-based nanomaterials and how this can be exploited to develop tailored materials towards specific applications.

## Abstract 2

Using the same starting materials but modifying the stoichiometry, we show that small variation in reaction conditions leads to changes in fluorescent behaviour of carbon dots, especially in the selective enhancement of overlapped fluorescence bands.

## Graphical abstract

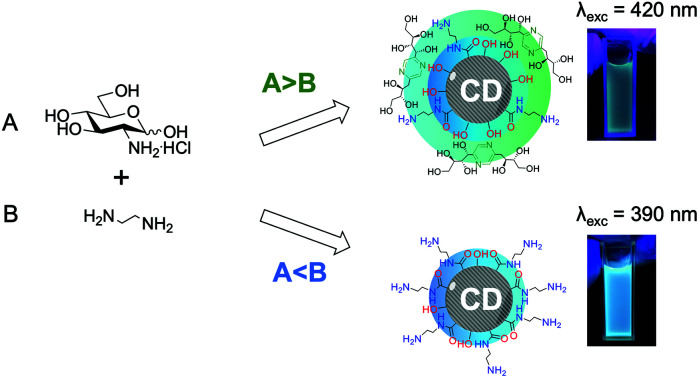

## Introduction

The growing field of nanotechnology has had a major impact on many research areas such as engineering, electronics, energy, environment, biology, and medicine.^1,2^ In this context, photoluminescent non-toxic and functional nanomaterials that can be easily prepared and tailored to specific applications are of great interest.^3–5^ Among the different types of luminescent nanodots, fluorescent carbon dots (CDs) have recently been described as a new class of carbon-based fluorescent nanomaterials with semi-spherical morphology, and unique optical and physico-chemical properties.^6–9^ Due to their tuneable photoluminescence, chemical inertness, high water solubility, low cost of fabrication and very low cytotoxicity,^10–12^ these materials have found applications in many research areas such as gene delivery,^10,13,14^ cell imaging,^12^ metal sensing,^11,15^ photo-catalysis,^16^ photosynthesis augmentation^17^ and photovoltaics.^18,19^

CDs have been produced from a range of organic starting materials including carbohydrates,^8,20^ amino acids^21–23^ and other small molecules^24^ using an array of methods such as thermal decomposition, chemical oxidation and hydrothermal oxidation under autoclave and microwave-assisted conditions. The resulting carbon dots have appreciable fluorescence quantum yields, different emission profiles and response to chemical environments such as pH detection capabilities.^25,26^

Unlike conventional inorganic quantum dots, CDs behave as nanoscale assemblies of fluorophores,^27,28^ with associated large Stokes shifts.^29–33^ Therefore, the fluorescent properties of the CDs are dependent on the molecular structure of the assembled components and surface defects. Further, these properties are tuned by the formation of CD microstructures,^34,35^ which in turn depend on the chosen synthetic strategy.^36^

One of the biggest challenges facing the field is the development of robust and reproducible synthetic strategies that lead to well characterised materials, while obtaining improved fluorescence quantum yields and distinct emission profiles.^37,38^ Despite many recent developments in this rapidly evolving field,^29,30,35,39,40^ a fundamental understanding at a molecular level of their formation mechanism, chemical structure and how these parameters are correlated to their photoluminescence (PL) properties is still lacking due to their complex physical structure. Consequently, most CDs syntheses are a result of serendipity, rather than rational design.^8,41^ Thus, understanding the factors that control their emission properties is essential for the development of tailored nanomaterials for bespoke applications.

Most studies to date focus on modifying the type of reagents, *e.g.* starting materials and/or addition of doping agents.^40,42,43^ Indeed, seminal efforts in this area have shown, for instance, that modulation of the optical properties of CDs can be achieved by the synergetic hybridisation of surface functional groups,^44–47^ heteroatom-doping^48–51^ or confined graphitisation enlargements of the core.^52–54^ Whereas the fluorescence quantum yield can be enhanced by maintaining structural homogeneity,^55^ monodispersity^56^ or surface protection towards environmental perturbations.^57,58^ Moreover, some reports have demonstrated the coexistence of multiple fluorescence centres within nanoparticles, each with different luminescent properties, thus adding a new strategy to optimise the overall optical performance.^59,60^

Efforts from our group to develop water-soluble fluorescent carbon-based nanoprobes for biological applications with tuneable fluorescent properties have shown that even small changes in the choice of starting materials can significantly change the resulting CD structure and physicochemical properties.^7–9,44^ For example, replacing the nature of the N-containing agent from TTDDA^9^ with an amino acid^8^ or aryldiamine^20^ in sugar-based CDs affords the nanoparticles with different fluorescence maxima. However, despite the evident significant effects in the resulting CDs, little attention had been given to study these effects in a systematic manner. Herein, we demonstrate how small changes in reaction conditions using the same common starting materials can be used to generate a range of CDs with tuneable photochemical and physical properties. Analysis of the structure, composition and optical properties of the materials helped us to identify distinct surface emission domains which are responsible for the material's optical properties and observed pH response.

## Results and discussion

Following on from our initial observations and to investigate the impact of reaction conditions in the final structural composition and physical characteristics of CDs, a modified three-minute microwave-assisted synthetic protocol^9^ using d-glucosamine hydrochloride (GlcNH_2_·HCl, 1) as the carbon precursor and ethylenediamine (EDA, 2) as a surface passivating agent, with varying stoichiometric ratios of reagents 1 : 2 from 3 : 1 to 1 : 12, was investigated (Table 1). This systematic study generated a library of CDs exhibiting different compositions as determined by NMR (Fig. S1†).

**Table 1:** Variations of stoichiometry in the synthesis of CDs and the CD : DOFZ ratios obtained from the ^1^H NMR resonance integrals – corresponding resonances for CD and DOFZ obtained at *δ* 3.2, 8.6 and 8.4 ppm, respectively

Sample	Stoichiometry of 1 : 2 (equiv.)	CD : DOFZ ratio
CD-1	3 : 1	0.2
CD-2	2 : 1	0.7
CD-3	1 : 1	1.1
CD-4	1 : 2	4.3
CD-5	1 : 3	6.8
CD-6	1 : 4	10.9
CD-7	1 : 6	11.9
CD-8	1 : 8	15.1
CD-9	1 : 10	20.8
CD-10	1 : 12	27.6

**Fig. 1:**
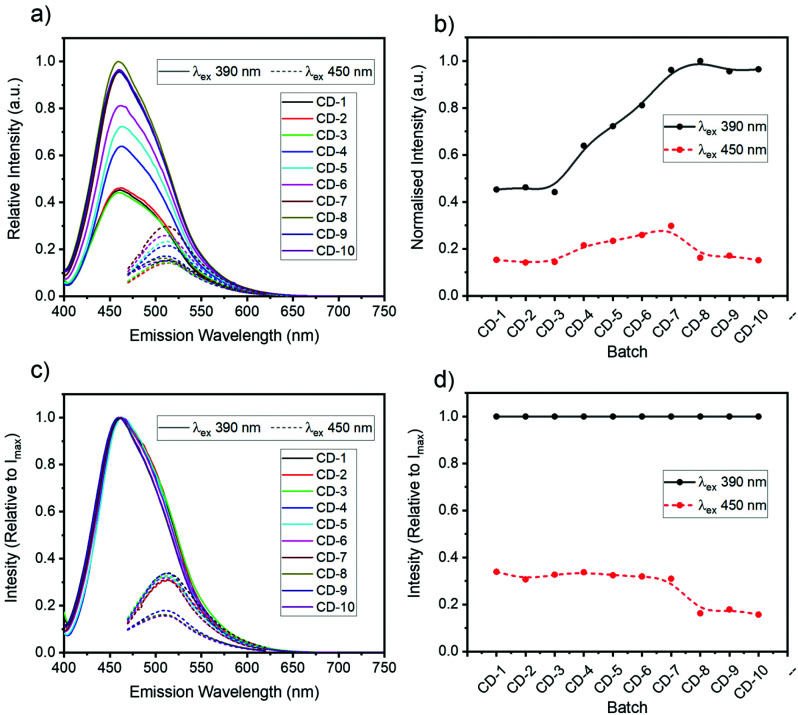
Fluorescence spectroscopy analysis of samples CD-1 to CD-10 excited at 390 nm (solid line) and 450 nm (dashed line): intensity normalised relative to CD-8 maximum (a and b) or normalised independently (c and d).

Fluorescence spectra of the all CDs (ranging between CD-1 to CD-10) revealed two main emission bands in the blue (460 nm) and green (510 nm) that were dependent and independent of the excitation wavelength (*vide infra*), respectively. The relative intensity of the blue and green fluorescence bands was also dependent on the initial d-GlcNH_2_·HCl : EDA (1 : 2) stoichiometry (Fig. 1). When excited with 390 nm light, CDs exhibited an attenuated green emission relative to the blue band. Interestingly, when excited at 450 nm, CDs made with equimolar or a higher ratio of 1 (*e.g.*CD-1 to CD-3, see Table 1), yielded enhanced green emission compared to CDs made with an excess of 2 (*e.g.*CD-8, Fig. 1). These results likely originate from the molecular and structural heterogeneity of multiple emissive molecules/centres present on the carbon dot structure and surface functionalisation. Having shown that the synthesized CDs exhibited different optical properties depending on the stoichiometry of reagents, CD-3 and CD-8 were chosen as representative case studies since all CDs shared common spectral features, albeit in different proportions.

### Composition and structure

**Fig. 2:**
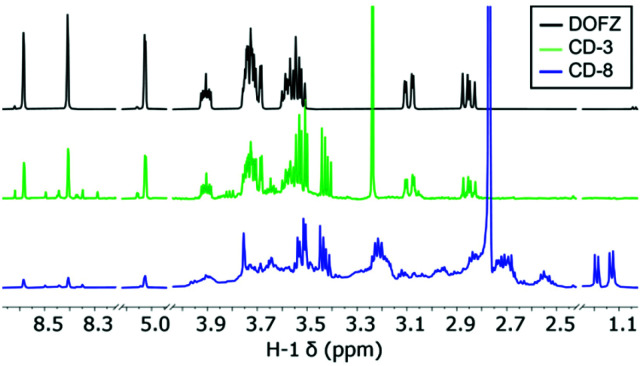
^1^H-NMR spectra for commercially obtained DOFZ, CD-3 and CD-8.

^1^H-NMR analysis was used to help elucidate the structure of the different nanomaterials after purification. Two sets of distinct signals associated to CD surface functionality at *δ* 1.15 and 3.45 ppm (Fig. 2 and S1†) could be assigned to sp^3^-protons likely linked to surface EDA, whilst aromatic peaks associated with pyrazine and other N-containing heterocycles were found between 8.6 and 8.3 ppm. Additionally, signals that could be attributed to surface-bound 2,5-deoxyfructosazine (DOFZ) were identified at *δ* 8.6, 8.4 and 5.0 ppm, especially for CDs that exhibited a more prominent green emission band (Table 1). Indeed, ^1^H-NMR analysis confirmed that when using an equimolar ratio of precursors 1 and 2, formation of DOFZ is favoured leading to CDs with DOFZ-related functionalities and an apparent enhancement in green emission. Conversely, as the ratio of EDA 2 was increased, the signals associated with DOFZ significantly reduced in intensity (see Fig. 2 and S1† and Table 1). Diffusion ordered spectroscopy (DOSY) NMR experiments of CD-3 (the CD which exhibited a significant green emission enhancement) showed that signals assigned to DOFZ had the same diffusion coefficient (3.8 × 10^−10^ m^2^ s^−1^) as signals attributed to the bulk CDs (see Fig. S2†), hence, suggesting that DOFZ is attached or adhered to the CDs. Moreover, the diffusion coefficients were correlated with the hydrodynamic radii of CDs using an approximation of the Stokes–Einstein equation, which correlated to an average radius of 0.86 nm for both CDs (Table S1†). Additionally, to confirm the presence of amino groups on the CD surface, CD-8 was reacted with succinic anhydride *via* carbodiimide conjugation, followed by extensive dialysis purification to yield acid-coated CDs. The presence of amino surface groups in CD-8 can be attributed to EDA-derived motifs generated during the CD surface passivation (Fig. S3†).

**Scheme 1:**
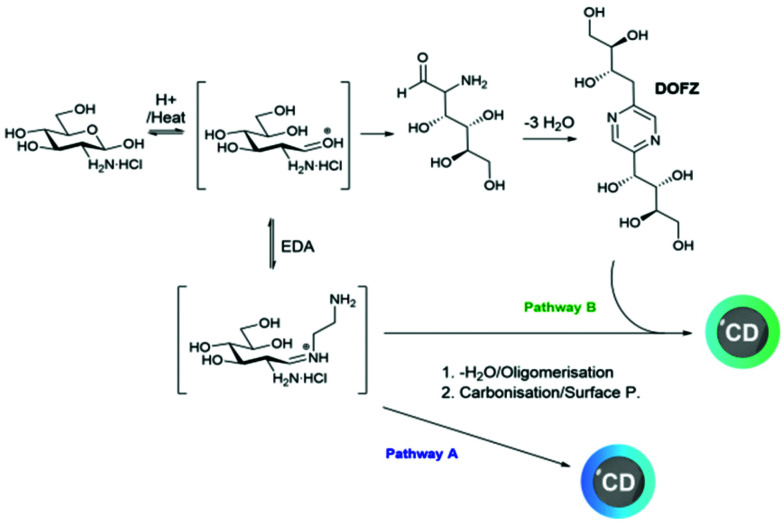
Proposed mechanism for the microwave-assisted synthesis of CDs from GlcNH_2_·HCl, 1 and EDA 2.

Previous work in our group has demonstrated that, in the synthetic pathway of CDs fabricated from 1 and aminated precursors, the aldehyde of the hemiacetal can react with the amine forming iminium intermediates prior to multiple steps of degradation/carbonisation^9^ (Scheme 1). Conversely, DOFZ is thought to be generated from 1,2-aminoaldose self-dimerisation^61^ and interestingly has been found as a surface component of other green-emitting CDs where 1 was reacted with a less nucleophilic amine source such as *m*-phenylenediamine.^20^ DOFZ is not inherently fluorescent at wavelengths longer than 380 nm (Fig. S4†), however its presence in CDs with enhanced green emission, suggests those materials undergo a different synthetic pathway that leads to the formation of alternative functional groups, which results in additional green fluorophores. It is likely that in the presence of an excess of EDA 2, sugar dimerization is suppressed and pathway A is preferred (Scheme 1). In instances when the concentration of sugar 1 is higher or equal to that of the diamine, the nucleophility of the amine doping agent can bias the reaction pathway. It is interesting to note that in the case of EDA 2 or *m*-phenylenediamine,^20^ pathway B where DOFZ is formed as intermediate, leads to apparent green emissive fluorophores, while using TTDDA^9^ or β-aminoacids^7^ at similar ratios yields predominantly blue emitting CDs.

**Fig. 3:**
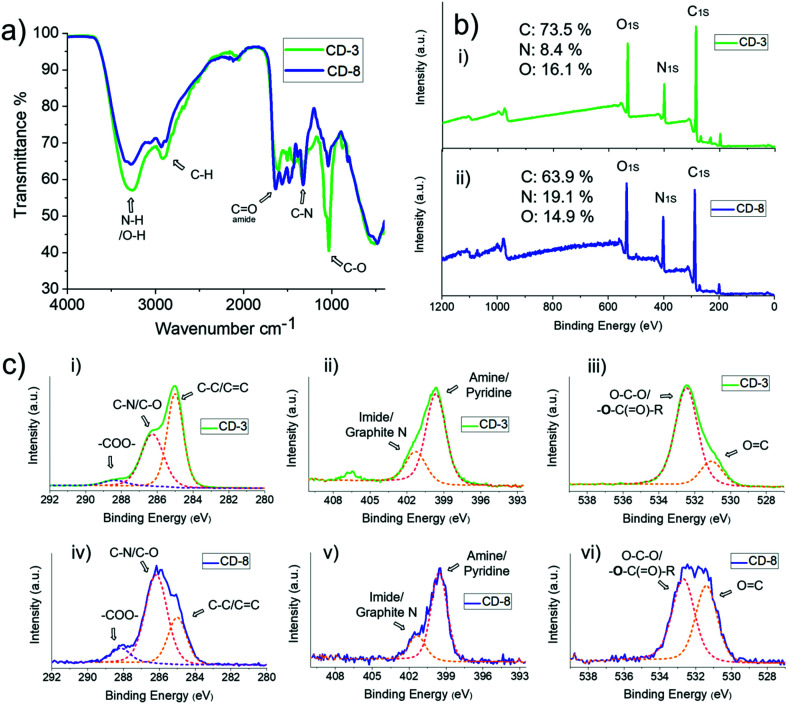
(a) FTIR, (b) XPS and (c) high-resolution XPS with deconvoluted C 1s, N 1s and O 1s signals of CD-3 and CD-8.

In order to study the physicochemical design of the CDs, Fourier-Transform Infrared Spectroscopy (FTIR) and X-ray Photoelectron Spectroscopy (XPS) were employed to interrogate the chemical and functional group composition of CDs, and X-Ray Diffraction (XRD), Atomic Force Microscopy (AFM) and High-Resolution Transmission Electron Microscopy (HR-TEM) analyses were performed to confirm the size and crystalline carbon structure of the nanoparticles. The presence of –OH/amide groups, which are most likely on the surface, was confirmed by analysis of the FTIR spectra displayed in Fig. 3(a), where the magnitude of the O–H/N–H stretch (3271 cm^−1^), C–N stretch (1325 cm^−1^), C–O stretch (1035 cm^−1^) and amide C=O stretch (1640 cm^−1^) were observed to vary between CD-3 and CD-8. For instance, a higher ratio of amide/imine/amino groups is present in CD-8 which can be correlated with the higher amount of EDA 2 used during the synthesis. Similarly, the XPS survey (Fig. 3(b)) indicates the presence of predominantly C, O and N heteroatoms in these materials. However, the N : C and O : C content varied with respect to the stoichiometry of the starting materials. As the ratio of 2 increased 8 times from CD-3 to CD-8, the N : C ratio increased by a factor of 2.62, while the O : C content had a discrete increase of 1.06. These findings further support a higher occupation of amide/amino enriched states on the surface of CD-8 than CD-3. High-resolution XPS survey provided additional information about the functionalisation of these heteroatoms within the structure of the CDs (Fig. 3(c)). The narrow C 1s spectrum can be fitted to deconvoluted signals centred at 285.0, 286.2 and 288.2 eV, which can be attributed to C–C/C=C, C–N and –COO–, respectively. While the peak in the O (1s) spectrum at ∼533 eV is likely to be O–C–O and the bold O in –**O**–C(=O)–R. Furthermore, the deconvoluted signal for O 1s lines clearly shows an increased prominence of the peak at 531.4 eV associated with C=O groups in CD-8 compared to CD-3, reflecting the greater presence of electron-withdrawing groups such as amides. The deconvolution of the N 1s signal suggests equivalent ratios of N groups within the composition of both CDs, attributed to primary amines (399.5 eV) and imide/graphite N (401.4 eV).

It is established that CDs are graphitic-like entities,^62^ with a delocalised excited state and associated extended exciton Bohr radius, and therefore the incorporation of O and N atom containing functional groups will introduce defects. In turn, this will modify important excited-state properties such as the band-gap, and thus impact the fluorescence maxima. Graphite-like nitrogen^63^ seen in XPS N 1s (401.4 eV), for instance, can reduce the energy required to exceed the optical bandgap in semi-crystalline CDs and hence, red shift the absorption/excitation of the CDs with respect to non-doped graphitic materials.^64,65^

**Fig. 4:**
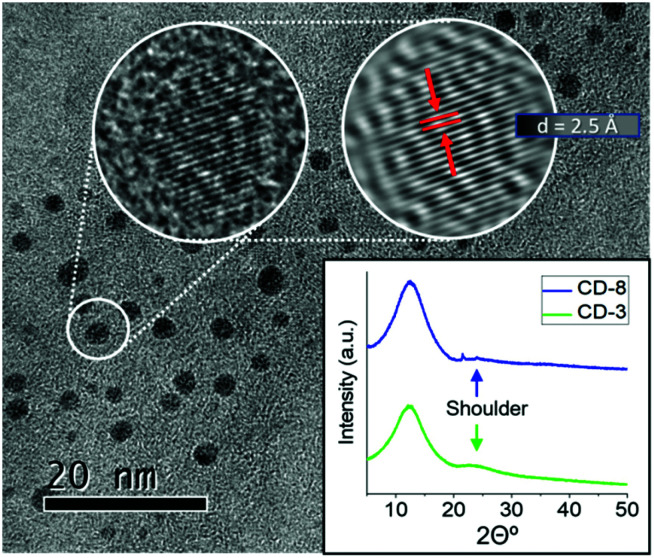
HR-TEM images of CD-8 and XRD analysis for CD-3 and CD-8.

The HRTEM imaging of CD-3 and CD-8 exposed the nanoparticles core structure to be semicrystalline in nature (Fig. 4 and S5†). Moreover, AFM image analysis altogether with the HRTEM results, revealed narrow size distribution for quasi-spherical particles with an overall size of about 1.4 nm (refer to ESI for full statistical analysis, Fig. S6–S10†). The XRD pattern of CD-3 and CD-8 showed a broad band at 12.5° and a small shoulder at 24.5° respect to 2*θ*. These diffractions correspond to a lattice inter-spacing of 0.68 and 0.34 nm for the plane (002) in graphite. The XRD results in conjunction with the *d*-spacing seen in HRTEM of 0.25 nm (corresponding to the (100) plane), suggest that the crystallinity observed in the CDs core is consistent with graphitic carbon encompassed in confined domains.^62,66^ The inhomogeneity in size distribution, however, is a reflection of the varying grades of crystallinity within the ensemble and the extent of graphitic domains, causing the broadening of the XRD signals. The crystallites size was hence estimated from the broadening of the XRD spectra using the Scherrer method with a dominant dispersity of around 1.4 nm (insertion in Fig. 4, S11 and Table S2†), which is in agreement to the hydrodynamic radius determined from the DOSY NMR and the size distribution determined by AFM. According HRTEM, AFM and XRD data, we can infer that those differences in crystallinity and core size are negligible between samples CD-3 and CD-8.

### Optical properties

**Fig. 5:**
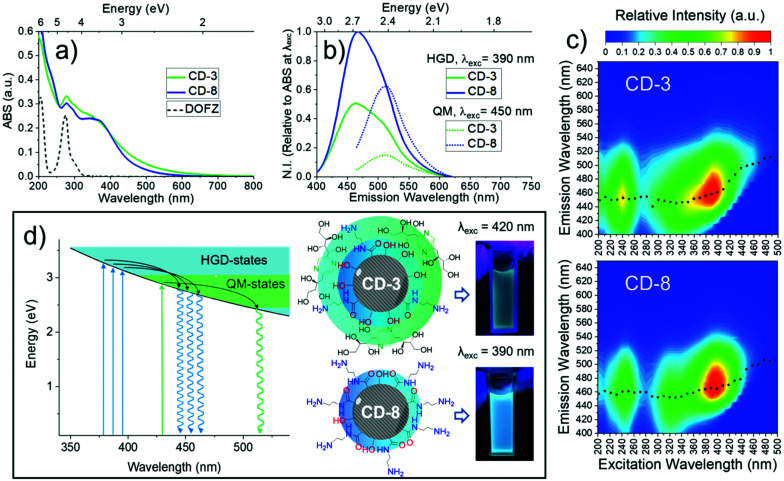
Optical properties of CD-3 and CD-8. (a) Absorption spectra. The DOFZ standard spectrum is included (dashed line). (b) Fluorescence spectra relative to absorption of each sample at the respective excitation wavelength. (c) 2D fluorescence spectra. Black dotted lines represent the maximum fluorescence wavelength at each excitation energy. (d) Schematic energy level diagram illustrating major non-radiative and fluorescent pathways associated with CD-3 and CD-8.

Steady state absorption spectroscopy was used to investigate the link between the CD composition and optical properties. The absorption spectra contain three major bands as evident in Fig. 5(a), including a strong absorption at wavelengths shorter than 260 nm associated with graphene domains, a more structured band centred at ∼275 nm attributed to DOFZ, and a final broad feature which spans into the visible, peaking at ∼350 nm. The overall absorption spectral shape can be attributed to a superposition of excited-state transitions which may originate from different chromophores, or multiple excited states of the same absorbers. This manifold of excited states can be explained in part by chemical alterations of aromatic conjugated systems/extended graphitic structures and structural heterogeneity.^64,67^

2D fluorescence spectra in Fig. 5(c) revealed two main features; deep-UV excitation (∼240 nm) was correlated with a blue (460 nm) fluorescence band. For longer wavelength excitation, a second (major) band is evident and exhibited excitation-dependent fluorescence and hence, multicolour emission with various coordinates in the chromaticity diagram (Fig. S12†). The maximum intensity of this band was associated with 390 nm excitation (a local maximum in the absorption spectrum). Photoexcitation with wavelengths longer than 405 nm resulted in a shifted fluorescence band peaking at 510 nm (correspond to green light) and was independent of the excitation energy.

In most of the 2D spectra, the bright blue emission obscures the green-shifted fluorescence band. However, the relative fluorescence intensity of both bands varied depending on the synthetic conditions (Fig. 1). Thus, the excitation-independent behaviour of the green band is most evident for excitation wavelengths >410 nm for CD-3 and >440 nm for CD-8.

In semicrystalline CDs, the effective conjugation length scales of the sp^2^ domains can localise electron–hole pairs, which strongly modifies the bandgap energy gap.^68^ Furthermore, graphene domains are altered by compositional/structural heterogeneity, giving rise to a continuous set of confined, heteroatom doped and surface/edge passivated sp^2^ domains, refer here as hybrid graphene domains (HGD). As each part of the hybrid structure has an associated different bandgap, it is therefore logical that this inhomogeneity is static (*e.g.* preserved) and the source of the observed excitation-dependent emission.

Turning now to the fluorescence which originates from excitation of 450 nm and longer wavelengths, we infer the excitation independent (*e.g.* Kasha like) behaviour originates from a chemically unique chromophore. It is likely, therefore, that these two photoluminescence centres co-exists in the CD structure and are structurally homogeneous and quasi-molecular (QM) in origin.^37^ The emission intensity of this centre is more pronounced for CD-3, and according to the FTIR, NMR and XPS analysis is due to the incorporation of DOFZ-related functionalities in the structure of the CDs.

Despite the apparent enhancement of the QM/HGD fluorescence in CD-3, the increased optical density at wavelengths >410 nm suggests that higher emission intensity does not correspond to an improved fluorescence quantum yield, but merely reflects greater absorbance as illustrated in Fig. 5(b) (see also Table S3 and Fig. S13†).

**Fig. 6:**
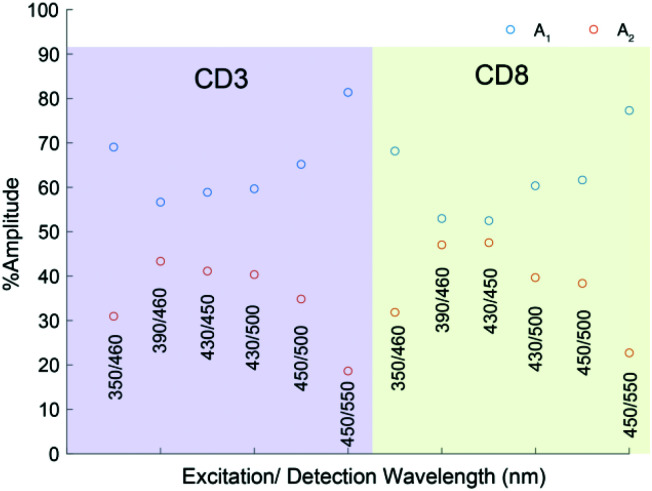
Normalised amplitudes of *A*_1_ and *A*_2_ as a function of different excitation and detection wavelength pairs for CD-3 and CD-8.

Time correlated single photon counting (TCSPC) studies interrogated the wavelength dependent fluorescence lifetimes of CD-3 and CD-8. These studies strengthened the hypothesis that two excited states contribute to the absorption between 350 and 450 nm. All data for CD-3 and CD-8 were globally fitted to a biexponential decay (convolved with the appropriate instrument response function), and only the normalised amplitudes allowed to change as either a function of the range of fluorescence wavelengths collected or CD sample. This returned two time constants of *τ*_1_ = 1.12 ± 0.06 ns and *τ*_2_ = 6.0 ± 0.2 ns attributed to fluorescence from QM and HGDs respectively, and the associated normalized amplitudes (*A*_1_ and *A*_2_) are given in Fig. 6. As these data could be accurately modelled with the same two exponential time constants and only the relative exponential pre-factor varying, it highlights the sensitivity of fluorescence to probing overlapping QM and HGD states.

The shorter lifetime component, *τ*_1_, dominates as the detected fluorescence wavelength is red-shifted from 450 nm to 550 nm, and thus assigned to QM domains. As the excitation and detection wavelengths are blue-shifted towards 390 and 450 nm respectively, the longer time constant associated with the HGD domains almost becomes equal in magnitude.

### Environmental response to cupric salts and pH

Experiments using cupric salts resulted in fluorescence quenching of both the blue (HGD) and green (QM) fluorescence bands (Fig. S14†). Such observations have been made before for CDs with Cu^2+^, and attributed to photoinduced electron transfer from carbon nanodots to copper ions.^44,69^ The efficiency of CD fluorescence quenching is far more notable for CD-8 compared to CD-3, which is correlated with the CD : DOFZ ratio (Table 1) – the DOFZ content of CD-3 is 15 times higher than CD-8. Therefore, most likely, if DOFZ is dominantly located within surface domains, it could increase the overall CD radii, and increase the net distance between HGD/QM domains and bulk solution. In turn, this would increase the overall distance to the electron accepting aqueous cupric ions, and thus reduce the probability of photoinduced electron transfer. This is also manifested in the AFM where the distribution of heights is broader for CD-3.

**Fig. 7:**
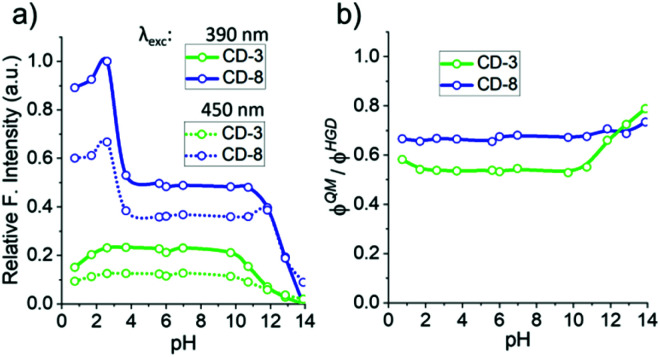
pH analysis of CD-3 and CD-8: (a) relative fluorescence intensity response to pH using an excitation wavelength at 390 nm and 450 nm. (b) Ratio of the emissions relative to the absorption of QM and HGD states using excitation wavelengths at 390 nm and 450 nm respectively.

A major advantage of the fluorescence arising from surface functionalisation is that its properties can be readily modulated by changing the surrounding chemical environment. Thus, the emission response of CD-3 and CD-8 was evaluated in a range of different pH (1–14) solutions (Fig. 7(a)). Interestingly, reversible quenching of the overall fluorescence was observed for both samples at alkaline pHs (>10). Further, a significant emission enhancement was observed for CD-8 at acidic pH < 4, while the fluorescence was quenched for CD-3 at pH < 2.5. This is likely due to the difference in surface functionalisation between the two nanoparticles, consistent with the Cu(ii) quenching results. It has been shown that surface trap-states associated with C=O, C=N, –OH and –NH functionalisation can alter the optical properties of HGD (linked to blue emission) upon protonation/deprotonation.^70,71^ Thus, the strong decrease in the fluorescence intensity observed for pH above 10 can be ascribed to the deprotonation of surface states which activates its trap behaviour leading to non-radiative processes.^72^

On the other hand, the dominant EDA 2 passivation in CD-8 suggests that protonation of amino groups has a significant effect on the fluorescence enhancement (pH < 4). This effect may be attributed to a significant contribution of protonated species in the ground state that can affect the excited state, as previously observed for other amino conjugated systems.^73^ Moreover, due to the length and flexibility of the EDA motif, it seems possible that protonation of the terminal amino group could lead to strong hydrogen bonding between the ammonium tail and surface carbonyl groups as has previously been reported.^74,75^ This proton-bridged cyclic structure could induce proton transfer in excited states to the carbonyl motif, and such a mechanism has been proposed for the preservation/enhancement of the fluorescent pathway in nanomaterials and aromatic molecules previously.^74,75^ Notably, CD-3 lacks EDA surface passivation, and the fluorescence yield at acidic pH is diminished relative to pH 7, which is likely associated with the inability to form the proposed cyclic networks.

To better understand the pH response observed, we then assessed the effect of pH changes on the individual chromophores found in CD-3 and CD-8. We screened the relative integrated emission intensity (*φ*) of samples in the pH scale, comparing the *φ* ratios of fluorescent bands that correlate to a quasi-molecular state and hybrid-graphene domains (*φ*^QM^/*φ*^HGD^, Fig. 7(b)). Between pH 2 and 10, the two fluorescent bands are equally affected by pH, keeping the *φ* ratio unchanged. However, over pH 10, the *φ*^QM^/*φ*^HGD^ ratio raises for both samples as result of a higher quenching contribution for *φ*^HGD^ than for *φ*^QM^. The effect is more significant for CD-3 and correlates to an apparent change to green colour in the overall emission at alkaline pHs.

## Conclusions

In summary, a range of CDs with distinct physico-chemical properties were prepared using the same common starting materials, (GlcNH2·HCl 1 and EDA 2), under three minutes of microwave irradiation, but modifying the stoichiometry of the reagents. It was found that small changes in reaction conditions, led to the modulation of composition, fluorescent behaviour and response to the environment in the CDs. Compositional and structural analysis of the different CD samples suggested that the reaction pathways followed during CD formation and surface passivation differed depending on reaction conditions, with the latter step being key to the observed differences, *e.g.* when an excess of 2 is used, hybrid-graphene domains are formed preferentially which leads to excitation-dependent blue emitting material; while if 1 is in excess, deoxyfructosazine derived functionalities are formed which leads to additional quasi-molecular fluorophores, which in turn yields an enhancement of the green-emissive band. Moreover, we demonstrate that the CDs can respond reversibly to changes in pH and that the response is due to different behaviour towards protonation/deprotonation events of the two distinct emission domains present within each CD. Our results highlight the importance of understanding the reaction pathways that lead to the formation of this carbon-based type of nanomaterials. Moreover, gaining better insights into how small changes in synthetic protocols lead to significant changes in the surface structure and composition of CDs, can be exploited to tune the physico-chemical properties of these exciting nanomaterials towards specific applications.

## Conflicts of interest

No conflict of interest to disclose.

## Supplementary Material

NR-014-D2NR01306A-s001
