# Supplementary material for: Small variations in reaction conditions tune carbon dot fluorescence
Source: Nanoscale. 2022 Mar 25;14(18):6930–40. doi: 10.1039/d2nr01306a (PMC9109711; doi:10.1039/d2nr01306a)
Supplement: NR-014-D2NR01306A-s001 [file NR-014-D2NR01306A-s001.pdf]

Electronic Supplementary Material (ESI) for Nanoscale.
This journal is © The Royal Society of Chemistry 2022

Small variations in reaction conditions tune carbon dot fluorescence

Support Information

Teodoro G. Millan,[a] Thomas A. Swift,[a] David J. Morgan,[b] Robert L. Harniman,[a] Benjamin Masheder,[c] Stephen Hughes,[c] Sean A. Davis,[a] Thomas A.A. Oliver,*[a] and M. Carmen Galan*[a]

[a]*School of Chemistry, University of Bristol, Cantock's Close, Bristol, BS8 1TS, UK.* [b]*Cardiff Catalysis Institute, Cardiff University, Park Place, Cardiff, CF10 3AT, Uk;* [c]*DST Innovations Ltd, Unit 6a Bridgend Business Centre, Bennett Street, Bridgend, CF31 3SH,*

* Corresponding Authors *E- mail: tom.oliver@bristol.ac.uk and m.c.galan@bristol.ac.uk*

CONTENT

1 General 2
1.1 Time-Correlated Single-Photon Counting (TCSPC) 2
1.2 X-ray Photoelectron Spectroscopy (XPS). 2
2 Synthesis of CDs 3
3 Characterization 3
3.1 1H Nuclear Magnetic Resonance Spectroscopy (NMR) 3
3.2 Diffusion ordered spectroscopy (DOSY) 4
3.3 Succinic Anhydride Conjugation. 5
3.4 Absorption and Fluorescence Spectra of DOFZ 5
3.5 PeakForce Atomic Force Microscopy (AFM) 6
3.6 High Resolution Transmission Electron Microscopy (HRTEM) 8
3.7 X-Ray Electron Diffraction (XRD) 8
3.8 Chromaticity Space 9
3.9 Quantum Yields 9
3.10 Cupric Ions Photo-quenching 10
References 11

1 General

^{1}H and ^{13}C (HSQC) NMR spectra were measured in D_2O at 500 MHz on a Varian spectroscope and their chemical shifts are quoted in parts per million (ppm). Fourier-transform infrared resonance was conducted on a Bruker ATR. Atomic force microscopy (AFM) was carried using a multi-mode VIII microscope with Nanoscope V control utilising a Fast-scan head unit whilst operating under PeakForce feedback control, using a SCANASYST-AIR-HR cantilever (Bruker, CA, USA) with a nominal spring constant of 0.4 N/m. Tip deconvolution using a Ti roughness sample (RS-12M; Bruker, CA, USA) revealed a tip radius of 4.98 nm 4 nm from the tip apex. High-resolution transmission electron microscopy (HRTEM) was conducted on a Jeol 2100F microscope with an accelerating voltage of 200 kV; for this characterization, a drop of the CDs in methanolic suspension (5mg/mL) was carefully applied to a 200 nm mesh carbon-coated copper grid and dried at ambient temperature. X-ray diffraction characterization was done using a Kappa Apex II diffractometer. Absorbance measurements were conducted on Cary UV-Vis 50 spectrophotometer in 3500 µL quartz cuvettes (ThorLabs). 2D Fluorescence measurements were obtained with a Perkin-Elmer LS45 in 3500 µL quartz cuvettes (ThorLabs) and samples were diluted prior analysis in order to avoid effects of self-absorption on the emission spectra (concentrations adjusted to an absorption lower than 0.1 at 390 nm). The conversion of the 2D fluorescent spectrum to x and y coordinates in the chromaticity space was achieved concerning the CIE colour matching functions. The approximation to sRGB from the chromaticity coordinates was done with PAL standard primaries using floating-point operations and a nonlinear correction using $\gamma = 2.2$.

1.1 Time-Correlated Single-Photon Counting (TCSPC)

An ultrafast oscillator (Coherent, Chameleon Ultra II) producing pulses at 80MHz was used to drive TCSPC experiments. The oscillator laser output was frequency-doubled in a b-barium borate crystal. The repetition rate of the laser was reduced to 3MHz using a Bragg cell driven by a high power radio frequency pulse generator (APE). These pulses were lightly focused into the sample (1cm pathlength cuvette) and the fluorescence detected at 90° relative to the laser excitation. The collected fluorescence was passed through a broadband polarizer set to magic angle and a band-pass filter to remove laser scatter and selectively acquire the sample fluorescence. The resulting light was focused onto an avalanche photodiode (IDQ, ID100-20-REG). The photon counts from the detector were acquired by a time-to- digital converter (Swabian Instruments, Time Tagger 20) and binned into histograms. TCSPC data were acquired in LabView. All data-analysis were performed in Matlab, where fluorescence decays were fit to a convolution of the fitted IRF function and two exponential decays.

1.2 X-ray Photoelectron Spectroscopy (XPS).

A Kratos Axis Ultra DLD system was used to collect XPS spectra using monochromatic Al $K\,\alpha$ X- ray source operating at 140 W (10 mA x 14 kV). Data was collected with pass energies of 160 eV for survey spectra, and 20 eV for the high-resolution scans with step sizes of 1 eV and 0.1 eV respectively. Samples were either pressed on to doubled sided Scotch tape (type 665), or for viscous samples, spread on to a UV cleaned Si wafer. The system was operated in the Hybrid mode, using a combination of magnetic immersion and electrostatic lenses, and acquired over an area approximately 300 700 μm^2. A magnetically confined charge compensation system was used to minimize charging of the sample surface, and all spectra were taken with a 90° take of angle. A base pressure of 1 x 10^{-9} Torr was maintained during the collection of the spectra. Data were analysed using CasaXPS (v2.3.23) after subtraction of a Shirley background and using modified Wagner sensitivity factors as supplied by the manufacturer.

2 Synthesis of CDs

Chemicals were purchased and used without further purification. D-glucosamine hydrochloride (1.00 g, 4.63 mmol) was dissolved in distilled H_2O (20 mL) in a 250 mL round bottom flask. ethylenediamine (EDA, quantity depending on the experiment) was then added to the solution and stirred vigorously for 30 min to ensure homogeneity. The round bottom flask was then placed in a domestic microwave 700 W (Wilko's Homebrand) and the solution was reacted for 3 min. After the microwave synthesis, a viscous brown residue was obtained which was taken up in distilled H_2O (10 mL). The crude solution was purified via centrifuge filtration (MWCO of 10 kDa, 8500 rpm, 30 min). The supernatant was concentrated *in vacuo* (or lyophilised) to yield a viscous brown syrup. The sample was then taken through size exclusion chromatography using a Sephadex G-10 resin acquired from GE Healthcare Life Sciences. Finally, the CDs were dialysed for 24 hours replacing with pure water frequently, employing a 500 Da dialysis membranes (purchased from Sigma-Aldrich). The resulting solution was concentrated *in vacuo*.

3 Characterization

3.1 ^1H Nuclear Magnetic Resonance Spectroscopy (NMR)

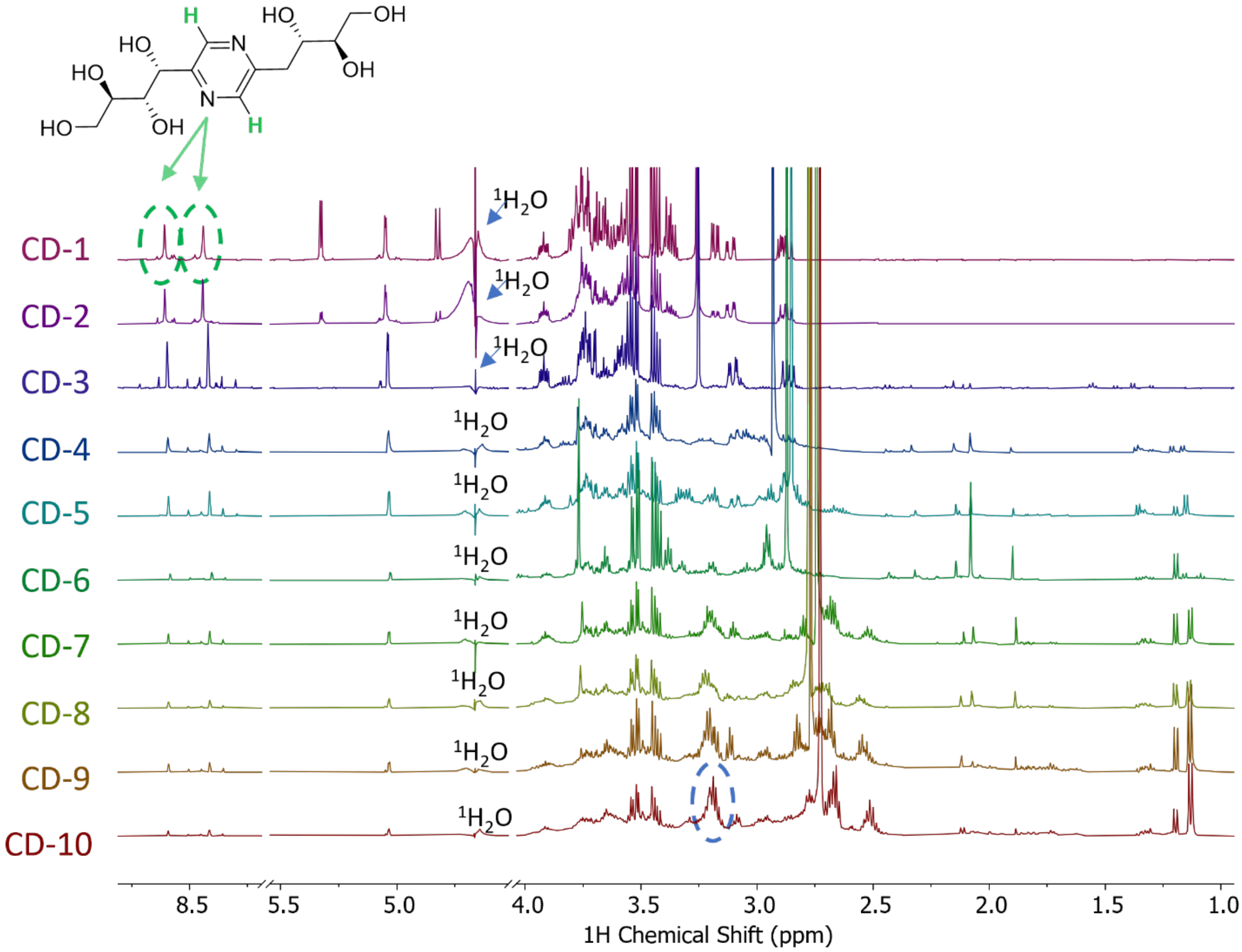

Figure S1: Hydrogen NMR resonances of synthesis of CDs varying the stoichiometry

3.2 Diffusion ordered spectroscopy (DOSY)

An indirect estimation of the hydrodynamic radius of the CDs (σ^{CD}) was possible using the Debye-Einstein equation and a correction for the diffusion coefficients [1]:

The Stoke Einstein equation tell us that.

$$D = \frac{K_b T}{c\,\eta\,\sigma}$$

Where *D* is the diffusion coefficient *Kb* is the Boltzmann constant, *T* is the temperature, *c* is a parameter that approaches 6 as the hydrodynamic radius (σ) reaches 1 nm and η is the viscosity of the solvent.

The estimation of σ^{CD} was calculated using a simplified relationship from the DOSY NMR (which probes the diffusion coefficient for each of the components of the 1H NMR spectrum), relative to the signal of ^1H_2O (D^w, 2.3×10^{-9} m^2/s, σ^w, 0.1375 nm) and considering the resonances that correlate to the CDs (table 1). This allows us not only to simplify the relationship of the Stoke-Einstein equation but also to ignore differences of viscosity in the sample.

$$\sigma^{CD} = \frac{D^w}{D^{CD}}\sigma^w \quad (1)$$

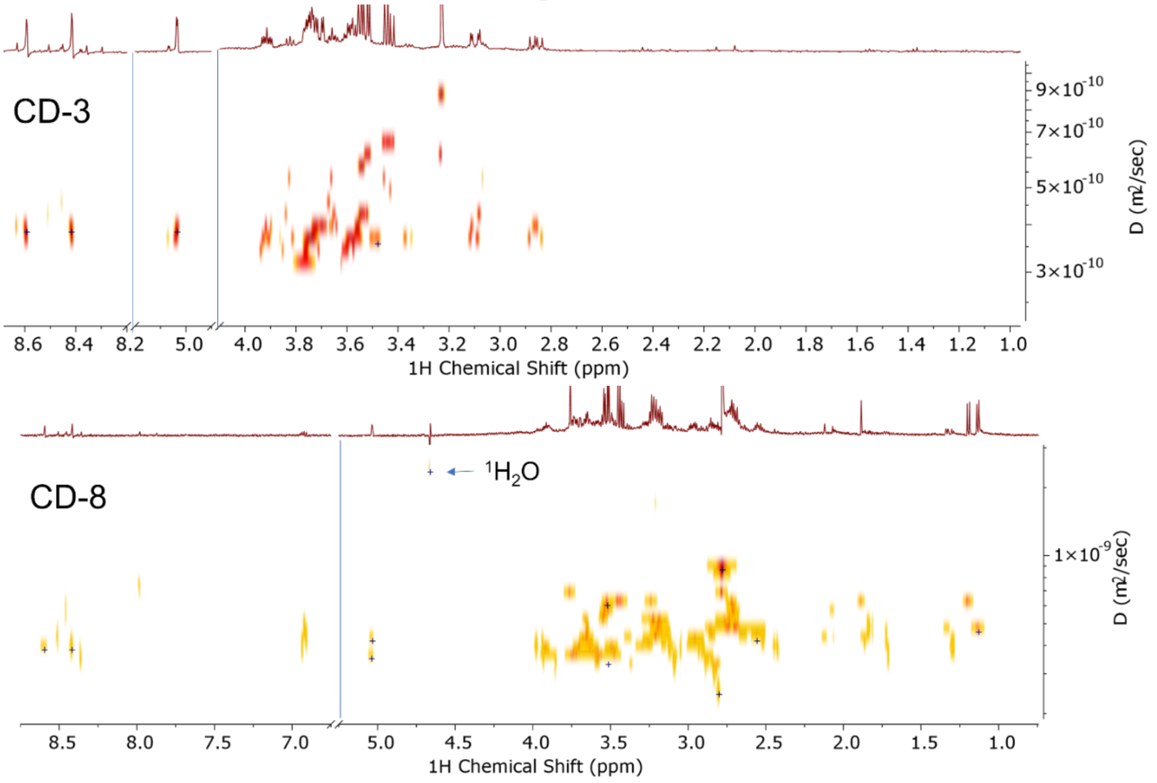

Figure S2: 1H DOSY NMR of **CD-3** and **CD-8**. The blue lines denote a crop in the spectra at different chemical shifts

Stoke-Einstein method				
	**CD-3**		**CD-8**	
δ (ppm)	D^{CD} (× 10^{-9} m^2/s)	σ^{CD} (nm)	D^{CD} (× 10^{-9} m^2/s)	σ^{CD} (nm)
8.6 - 3.9	0.38	0.83	0.38	0.83
3.8 - 3.4	0.35	0.90	0.33	0.96
3.3 - 1.0			0.40	0.79

Table S1: Hydrodynamic radius of CDs. Estimation relative to the water signal using the equation (1).

3.3 Succinic Anhydride Conjugation.

Conjugation of succinic anhydride (SA) on the **CD-8** was possible via carbodiimide coupling. For the hydrolic cleavage of SA, 100 mg (1 mmol) of SA were dissolved in water in a 100 ml round bottom flask. Subsequently, 440 mg 1-ethyl-3-(3-dimethylaminopropyl)carbodiimide (EDC, 2.88 mmol) and 390 mg N-hydroxysuccinimide (NHS, 3.45 mmol) were added to the solution and sonicated. Then, 200 mg of the lyophilized **CD-8** sample was added and the solution and stirred vigorously overnight. The solution was then passed through a size exclusion column using a Sephadex G-10 resin and reduced *in vacuo*.

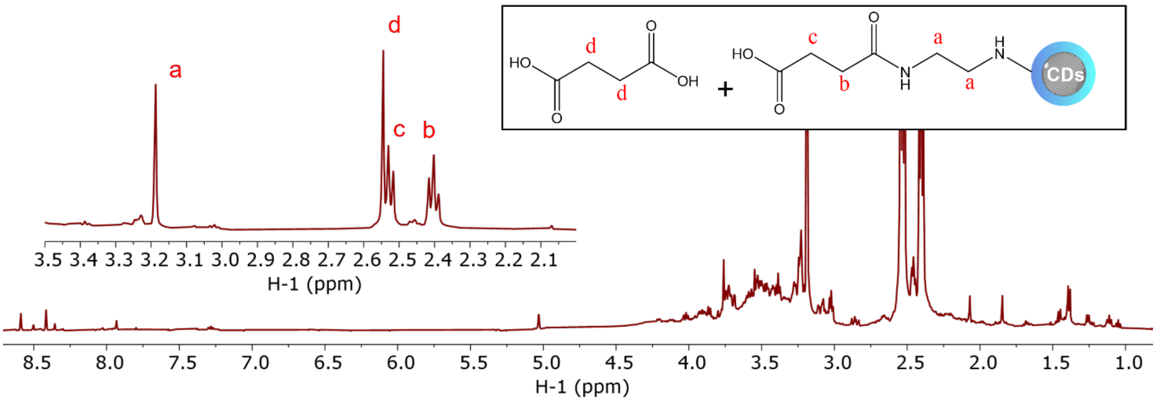

Figure S3: ^{1}H NMR - Succinic Anhydride conjugation on **CD-8**

3.4 Absorption and Fluorescence Spectra of DOFZ

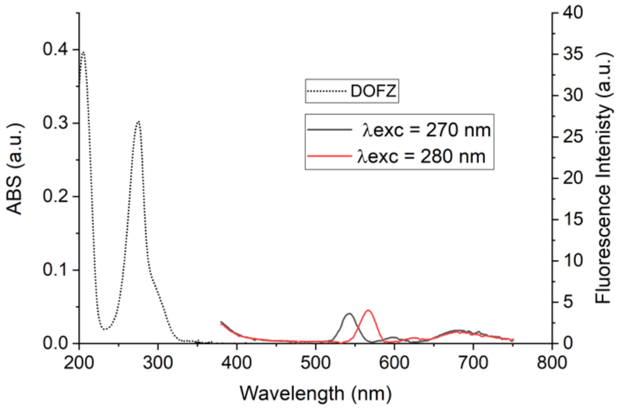

Figure S4: Absorption (dashed line) and emission spectra (straight lines) of DOFZ.

3.5 High Resolution Transmission Electron Microscopy (HRTEM)

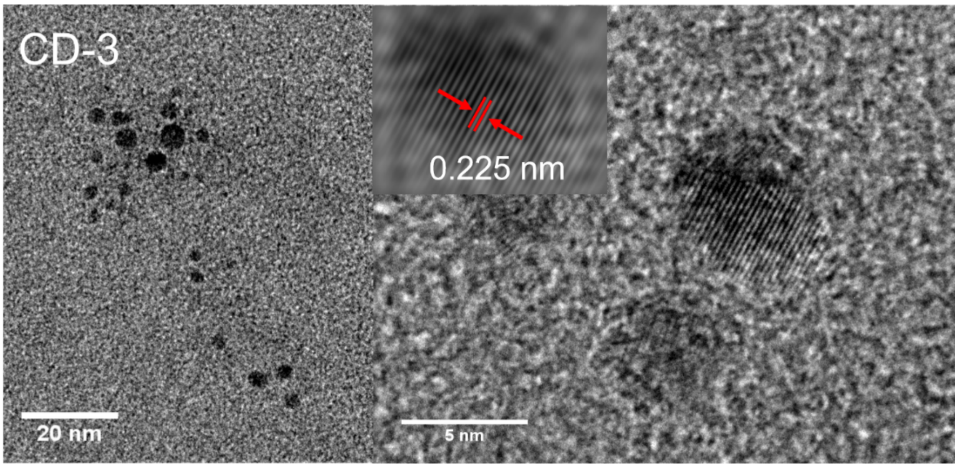

Figure S5: HRTEM images of sample **CD-3.**

3.6 PeakForce Atomic Force Microscopy (AFM)

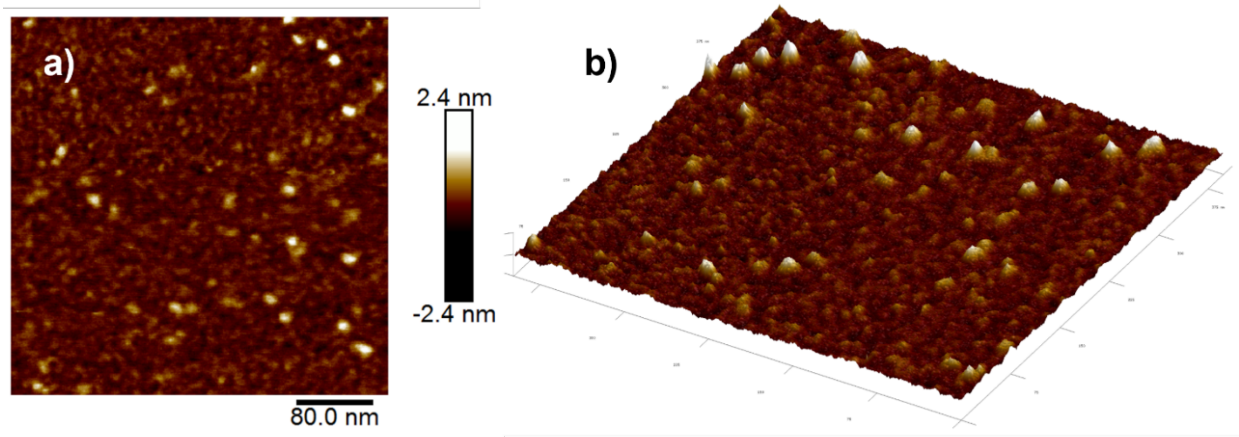

Figure S6: AFM of **CD-3**. Image on view in 2D (a) and 3D (b).

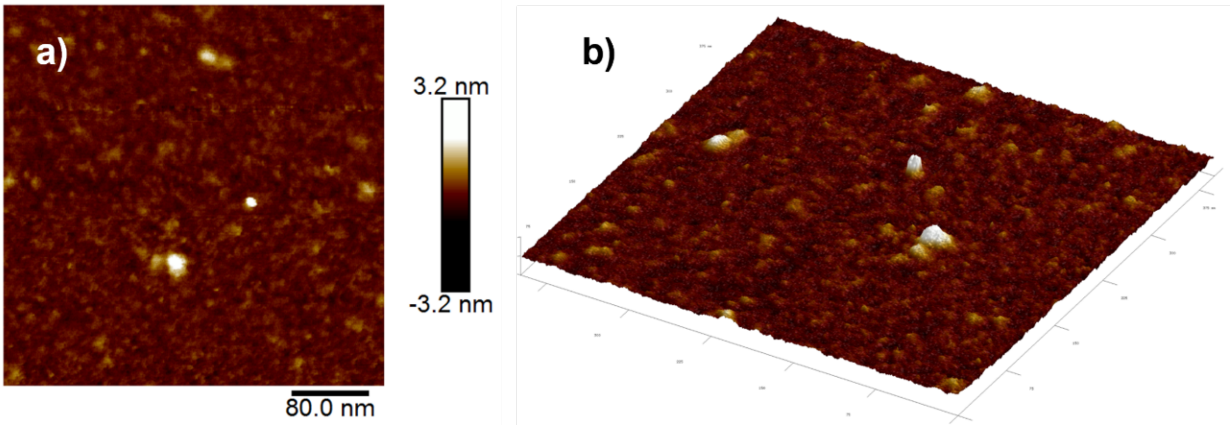

Figure S7: AFM of **CD-8**. Image on view in 2D (a) and 3D (b).

3.6.1 Statistical Analysis of CDs Measures

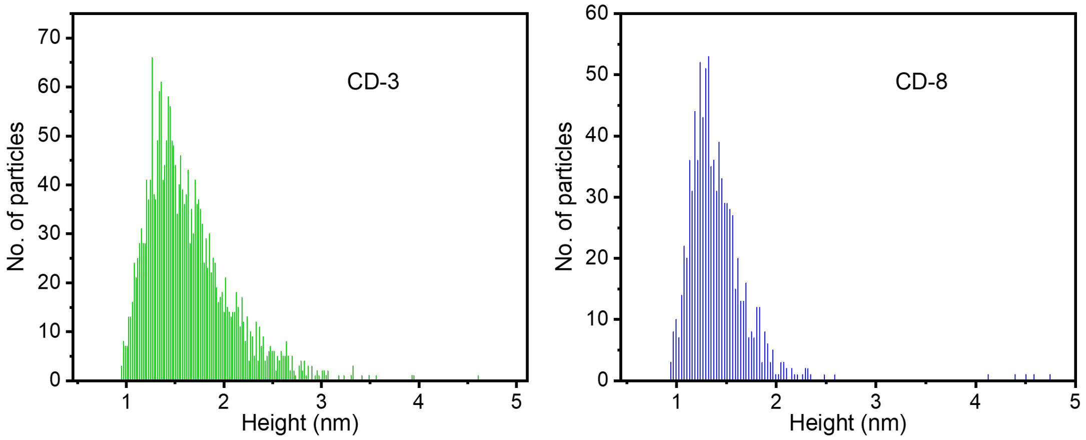

Figure S8: Height distribution of **CD-3** and **CD-8**.

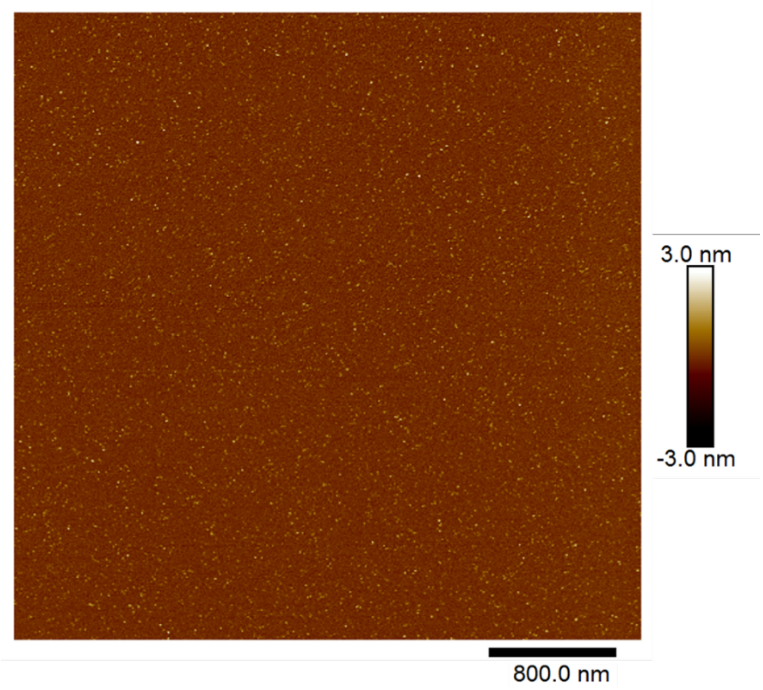

Figure S9: AFM image used for statistical analysis measurements of **CD-3**.

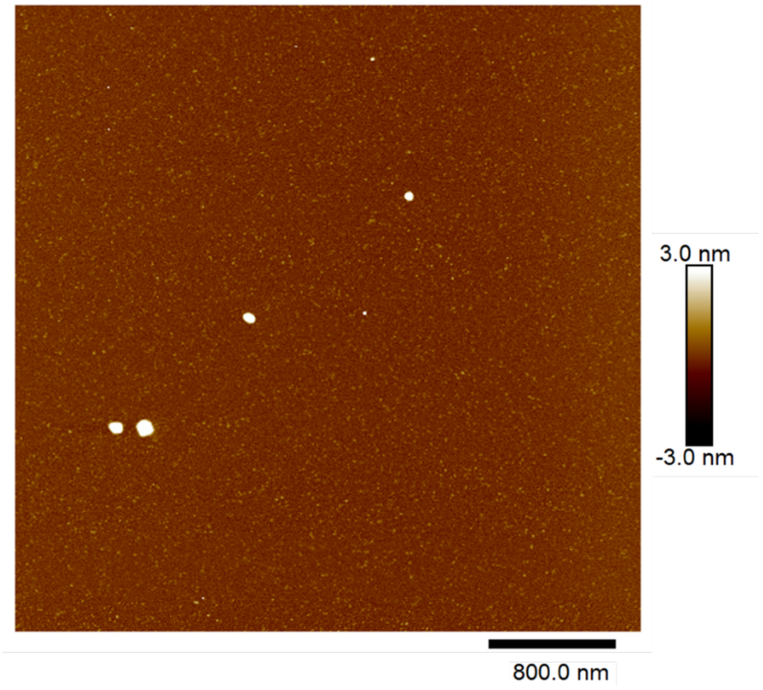

Figure S10: AFM image used for statistical analysis measurements of **CD-8**.

3.7 X-Ray Electron Diffraction (XRD)

The crystallite size (*D*) of samples **CD-3** and **CD-8** was determined based on the broadening of the XRD signals using the Scherrer equation.

The Scherrer equations tell us that:

$$D = \frac{k\lambda}{\beta d\ cos\theta} \quad (2)$$

Where *D* is the crystallites size, λ = 1.5418 Å is the wavelength of the X-ray source, *k* = 0.9 is the shape factor and *βd* is the peak broadening caused by the crystallite domain in radians. The *βd* has been approximated to the total broadening (βt) of the peak, which is determined by the full width at half maximum (FWHM). However, the βt of the XRD peaks is a contributed effect caused not only by the crystallites size broadening (βd) but also by the microstrain and the instrumental broadenings (βs and βi respectively). This is:

$$\beta t = \beta d + \beta s + \beta i$$

Hence, *βd* can only smaller or equal than the FWHM, which will imply that the estimation for *D* is only smaller than the real length of *D*.

Scherrer method			
Sample	θ (radians)	β_t (radians)	*D* (nm)
**CD-3**	0.108	0.101	1.378
**CD-8**	0.109	0.096	1.443

Table S2: Crystallites size (*D*) determined using the Scherrer method with equation (2)

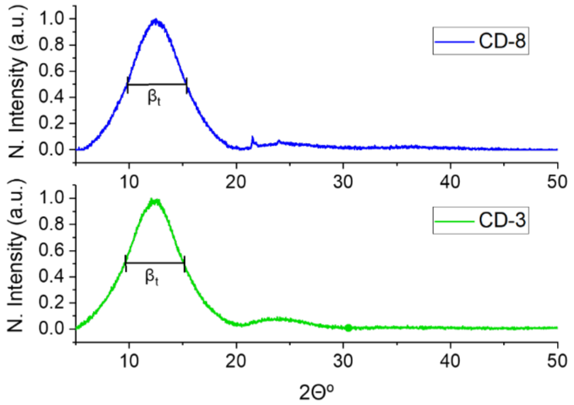

Figure S11: XRD spectroscopies of samples **CD-3** and **CD-8**. FWHM extracted from peak centred at 12.4° and 12.5° of 2Θ respectively.

3.8 Chromaticity Space

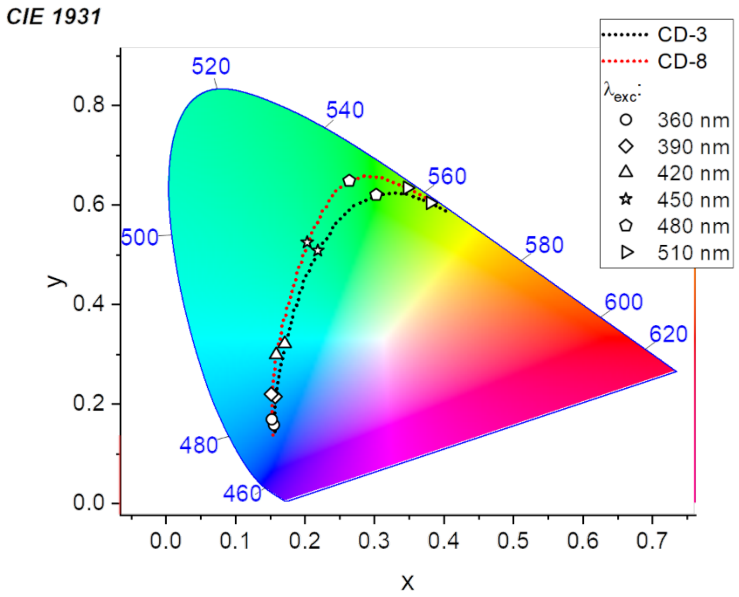

Figure S12: Chromaticity Space separation of samples **CD-3** and **CD-8** at different excitation wavelengths.

3.9 Quantum Yields

Quantum Yield (Φ) estimation was performed based on IUPAC protocol [2]. The spectral data measurements for CDs were conducted on both Perkin-Elmer LS45 and Cary UV-Vis 50 spectrophotometer in 3500 µL quartz cuvettes. The calculous was estimated at excitation wavelengths where a dominant hybrid-graphene-derived (HGD) state transition or a quasi-molecular (QM) state transition are seen, relative to quinine sulfate (in 0.1 M H_2SO_4) or Coumarin 153 (in pure EtOH) respectively. A $\Phi_{QuinineS.}$ of 60% and of $\Phi_{C.153}$ of 38% were considered.

Quantum Yields				
λ_{exc} (nm)	HGD		QM	
	Φ_{CD-3}	Φ_{CD-8}	Φ_{CD-3}	Φ_{CD-8}
350	0.019	0.081		
390	0.032	0.09		
430			0.016	0.051
450			0.01	0.027

Table S3: Quatum yields measurements for **CD-3** and **CD-8.**

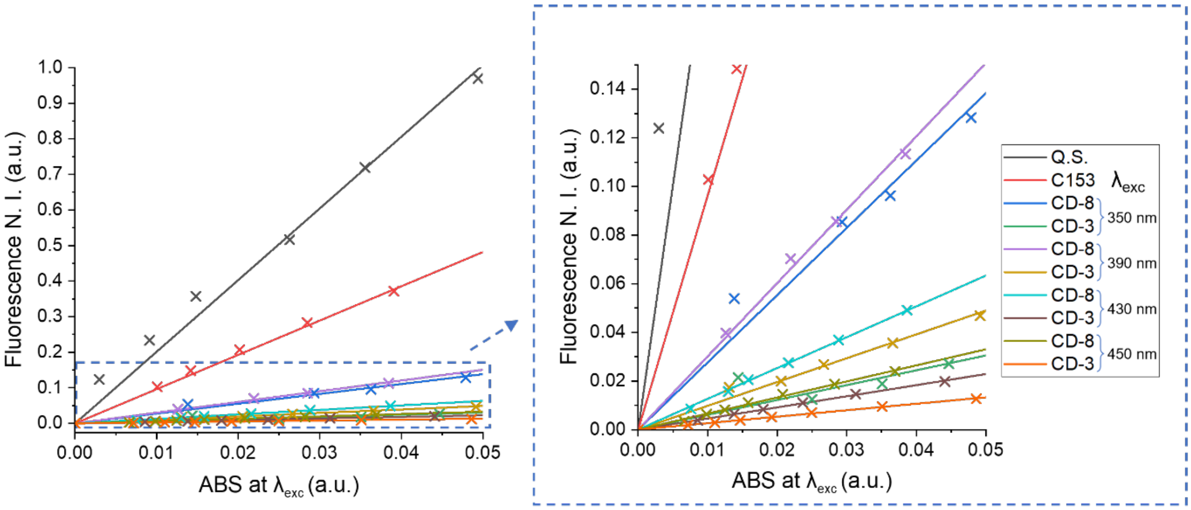

Figure S13: Standard Gradient Curves of CDs for QY determination

3.10 Cupric Ions Photo-quenching

The fluorescence of samples **CD-3** and **CD-8** was quenched with the addition of cupric ions at different concentrations. A 100 ml aqueous batch solution of **CD-3** was prepared to a concentration of 800 µg/ml. The solution was partitioned into 50 ml in separated round bottom flasks and 150 mg of copper (II) chloride were added to one of the partitions. Finally, serial dilutions were prepared from the two partitions to vary the concentration of cupric ions and keep the concentration of CDs. The same procedure was employed for sample **CD-8**.

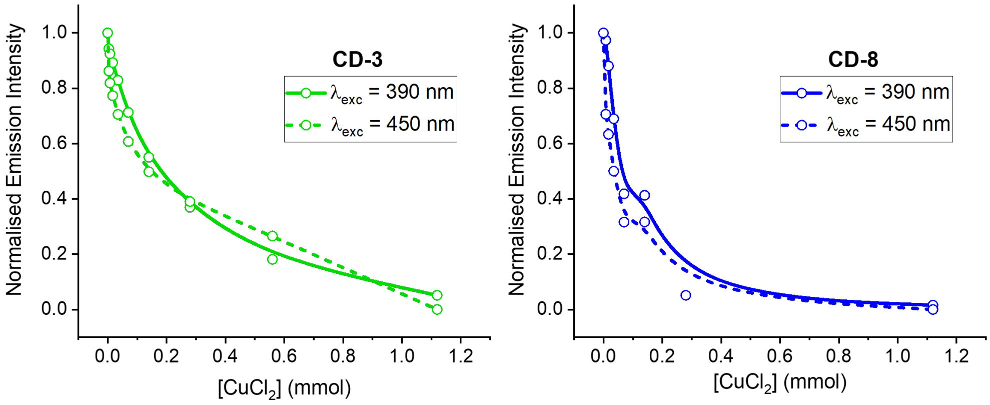

Figure S14: Fluorescence quenching of **CD-3** and **CD-8** upon addition of copper(II) chloride.

4 References

[1] Gabriele Canzi, Anthony A Mrse, and Clifford P Kubiak. Diffusion-ordered nmr spectroscopy as a reliable alternative to tem for determining the size of gold nanoparticles in organic solutions. The Journal of Physical Chemistry C, 115(16):7972–7978, 2011.

[2] Albert M Brouwer. Standards for photoluminescence quantum yield measurements in solution (iupac technical report). Pure and Applied Chemistry, 83(12):2213–2228, 2011.
